# Morphological changes of contralateral intervertebral foramen induced by cage insertion orientation after unilateral transforaminal lumbar interbody fusion

**DOI:** 10.1186/s13018-019-1121-1

**Published:** 2019-03-13

**Authors:** Kai Zhu, Shuaifeng Yan, Song Guo, Jinyu Tong, Cong Li, Jun Tan, Weiping Wan

**Affiliations:** 1 Department of Orthopaedic Surgery, Shanghai East Hospital, Tongji University School of Medicine, 150 Jimo Rd, Pudong New District, Shanghai, 200120 China; 2 Department of Radiology, Changzheng Hospital, Second Military Medical University, 415 Fengyang Rd, Shanghai, 200003 China

**Keywords:** Transforaminal lumbar interbody fusion, Intervertebral foramen, Cage, Segmental angle, Lumbar spinal stenosis

## Abstract

**Background:**

This study was performed to investigate the morphological changes of contralateral intervertebral foramen (IVF) based on computed tomography images of patients with lumbar spinal stenosis after unilateral transforaminal lumbar interbody fusion (TLIF) and to compare the influence of different orientation of cage insertion on these changes.

**Methods:**

This is a retrospective cohort study. Sixty-nine patients with lumbar spinal stenosis who had undergone single-level unilateral TLIF were retrospectively analyzed. The patients were divided into two groups according to the cage insertion orientation: the oblique group (o-group, 39 cases) and the transverse group (t-group, 30 cases). The morphological parameters of contralateral IVF were measured before and 6 months after the operation. Changes in these parameters were compared and analyzed between the two groups. The 6-month clinical outcomes of the two groups were also collected and analyzed.

**Results:**

There was a significant difference in the rate of increase in the segmental angle (*p* < 0.01) between the two groups, the mean value of segmental angle increased by an average of 29.08% ± 14.93% in the o-group and 48.63% ± 12.01% in the t-group. Overall, the posterior disc height had a significant positive correlation with the foraminal height and area. In the o-group, however, an increase in the segmental angle resulted in a decrease in the foraminal area. No significant difference in clinical outcomes was found between the two groups.

**Conclusions:**

Compared with oblique cage insertion, transverse cage insertion could achieve greater restoration of segmental lumbar lordosis without decreasing contralateral foraminal dimensions.

## Background

Transforaminal lumbar interbody fusion (TLIF) was introduced by Harms and Rolinger in 1982 [1] and popularized by Harms and Jeszenszky D in 1998 [2]. Since then, its effectiveness and safety have been demonstrated in a large number of literature. The best indications for TLIF procedure include degenerative spondylolistheses, recurrent disc herniation, spinal stenosis with segmental instability, and discogenic back pain that is unresponsive to conservative treatment [3–5]. TLIF has many advantages over other infusion techniques, such as less blood loss, less nerve root retraction, and less destruction of the posterior elements. Furthermore, growing evidence has indicated that an additional effect of TLIF is contralateral indirect decompression. Min et al. [6] found that the clinical outcomes of contralateral indirect decompression via unilateral TLIF procedure were satisfactory. Iwata et al. [7] investigated the mechanism of this effect by detailed radiographic measurements on the contralateral intervertebral foramen (IVF); however, his study involved patients with spondylolisthesis and neglected the extra impact of intravertebral reduction on IVF morphology in the presence of spondylolisthesis. Similarly, Kim et al. [8] documented that the morphological parameters of contralateral IVF increased significantly after unilateral TLIF and that these changes might contribute to indirect decompression on the contralateral side; however, this study overlooked the effects of different cage location within the intervertebral disc space (IVS).

In recent years, many studies on TLIF have been focused on exploring cage-related issues regarding the cage location [9], cage material [10], and cage shape [11, 12]. In contrast, few studies have considered the orientation of cage insertion and its possible impacts on postoperative outcomes of TLIF. Our previous study [13] has demonstrated the efficacy and safety of transverse cage insertion but failed to make any comparison with other orientation of cage insertion. Hence, the main objective of the present study was to investigate the postoperative morphological changes of contralateral IVF of patients with spinal stenosis and to compare the effects of different orientation of cage placement on these changes.

## Materials and methods

### Subjects

**Fig. 1:**
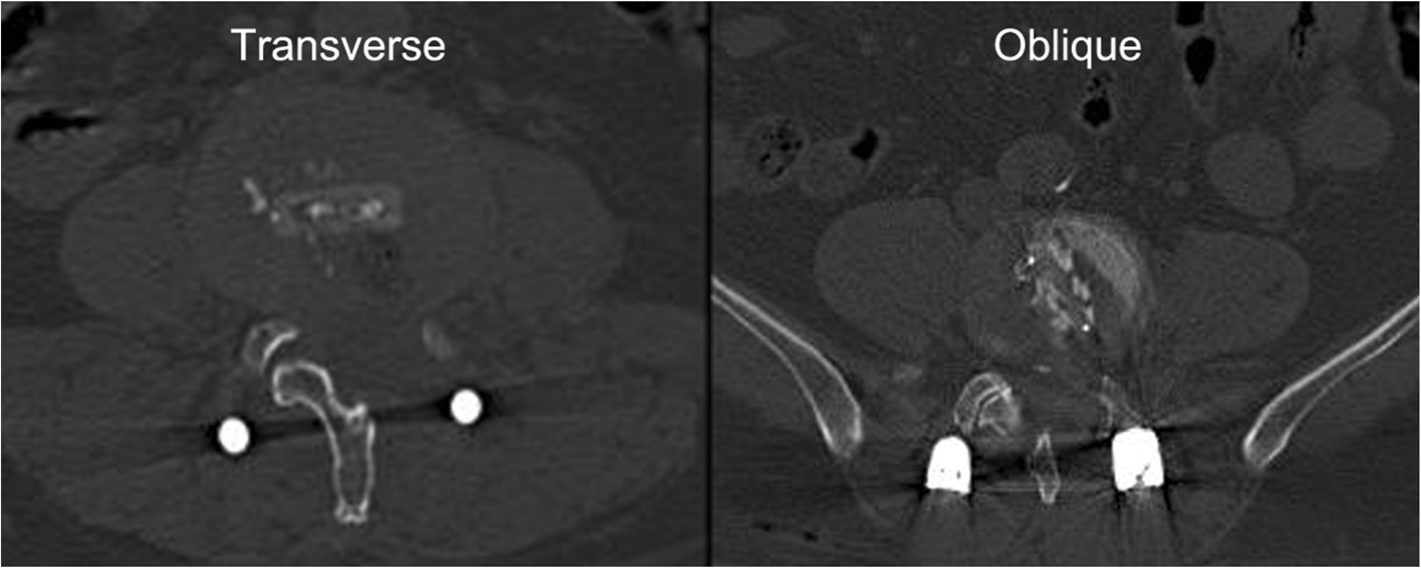
Two cage placement orientations

This is a retrospective cohort study that was approved by the Institution Review Board of East Hospital affiliated to Tongji University School of Medicine. Informed consent and protocols were obtained from all patients, which included details of the surgery including the mechanism of treatment, predictive outcomes, and potential risks and adverse effects. Between December 2014 and July 2017, a total of 120 consecutive patients in our department with degenerative lumbar spinal stenosis were retrospectively analyzed. Forty-one patients who had undergone bilateral TLIF or other surgical procedures were excluded from our study. To eliminate the impact of lumbar reduction on the morphological changes of IVF, ten patients with concurrent spondylolysis or spondylolisthesis were also excluded from our study. Finally, the remaining 69 patients who had undergone single-level unilateral TLIF were included in this study. The included patients consisted of 29 males and 40 females, aging from 38 to 75 years (average 49 years). They were divided into two following groups according to the cage insertion orientation used (Fig. 1): the oblique group (o-group, 39 cases) and the transverse group (t-group, 30 cases). The choice of cage insertion orientation was based on different time periods, and no other selection criteria were used in the study. Oblique cage insertion method was performed in early stage during the study period, while transverse cage insertion method was performed during the more recent years. The detailed demographic and clinical characteristics are listed in Table 1. The interbody cage used in TLIF was the bullet-shaped Concorde cage (DePuy Spine, Raynham, MA, USA).

**Table 1:** Patient demographics in the oblique and transverse groups

	Oblique (*n* = 39)	Transverse (*n* = 30)	*p* value
Sex (male/female)	18/21	11/19	0.429
Age (years)	50.41 ± 8.34	47.40 ± 6.10	0.101
BMI (kg/m^2^)	22.97 ± 1.60	23.66 ± 1.39	0.067
Fusion Levels			0.851
L3–L4	4	2	
L4–L5	28	23	
L5–S1	7	5	
Cage height (mm)			0.428
10	2	4	
11	22	16	
12	15	10	

*BMI* body mass index

### Surgical procedure

The patients were placed in the prone position on a radiolucent table under general anesthesia. A posterior midline incision was made, the unilateral paravertebral muscles were stripped, ipsilateral pedicle screws were implanted, the inferior articular process and the upper half of the superior articular process (SAP) were removed, and the affected nerve root was fully decompressed. After total discectomy and cartilage endplate resection were performed, a single cage filled with autologous bone was inserted and positioned as close to the center of the IVS as possible (crossing the midline). Two cage insertion orientations were used in the procedure: oblique and transverse. Transverse cage insertion was achieved by the method described in our previous study [13]. To obtain a solid interbody fusion, the IVS was filled with autologous bone chips harvested from the removed lamina and articular process. In the end, percutaneous pedicle screw fixation was performed on the contralateral side. The intervertebral compression through the screw–rod system was not performed during the procedure. All cases were operated by the same surgical team. After surgery, a lumbar back brace was recommended for over 6 weeks.

### Radiographic assessment

A 64-row multidetector computed tomography system (Brilliance, Philips Medical Systems, Cleveland, USA) was used in all patients before and 6 months after surgery. Three-dimensional reconstruction and all the measurements were performed with Impax PACS (version: 6.5.3.3009, Agfa Healthcare, Belgium). CT scanning of the same patient was performed under identical conditions before and after the operation.

### Evaluation items

The entrance zone of the contralateral IVF was studied. It was defined as the space between the medial edges of the superior and inferior pedicles in the sagittal plane.

*The disc-to-facet distance (D-F)*: The vertical distance between the apex of SAP and posterior margin of the involved disk in the sagittal plane.

*Foraminal height (FH)*: The maximum distance between the inferior margin of the pedicle of the superior vertebra and the superior margin of the pedicle of the inferior vertebra.

*Posterior disc height (PDH)*: The distance between the upper and lower endplates of the involved disc at the posterior margin.

*Segmental angle (SA)*: The angle between the upper and lower endplates of the involved disc in the sagittal plane.

*Foraminal area (FA)*: In the sagittal plane, FA is bounded by the surfaces of the upper and lower pedicles, the surface of intervertebral disk anteriorly and the surface of the ligamentum flavum posteriorly.

**Fig. 2:**
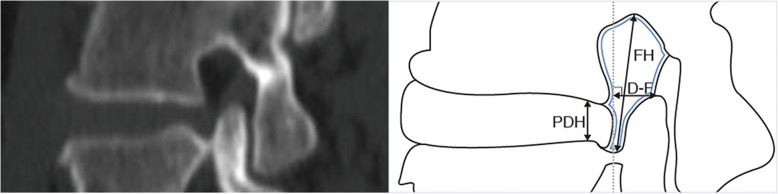
Diagram showing the measurements made on the disc and intervertebral foramen. D-F (disc-to-facet distance), the vertical distance between the apex of the superior articular process and posterior margin of the involved disk; FH (foraminal height), the maximum distance between the inferior margin of the pedicle of the superior vertebra and the superior margin of the pedicle of the inferior vertebra; PDH (posterior disc height), the distance between the posterior ends of the upper and lower endplates at the disc space; FA (foraminal area), area surrounded by blue solid line

All of the above parameters (Fig. 2) were measured by an orthopedic spine surgeon with more than 10 years of experience in spine surgery and by a radiologist who specialized in the musculoskeletal system, respectively. The mean values of the two measurements were used.

### Statistical analysis

Given that the sample size was small, non-parametric tests were used for statistical analysis. The Mann–Whitney *U* test was used for unpaired data, and the Wilcoxon signed rank test for paired data. Multiple linear regression analysis was used to adjust for the potential confounding variables. Correlations among various parameters were analyzed using Spearman’s correlation coefficient. Statistical analysis was carried out using SPSS 21.0 software (SPSS Inc., Chicago, IL, USA); *p* < 0.05 was considered as significant. The data are presented as means ± standard deviation.

## Results

### Change in various parameters of the contralateral IVF after surgery

**Fig. 3:**
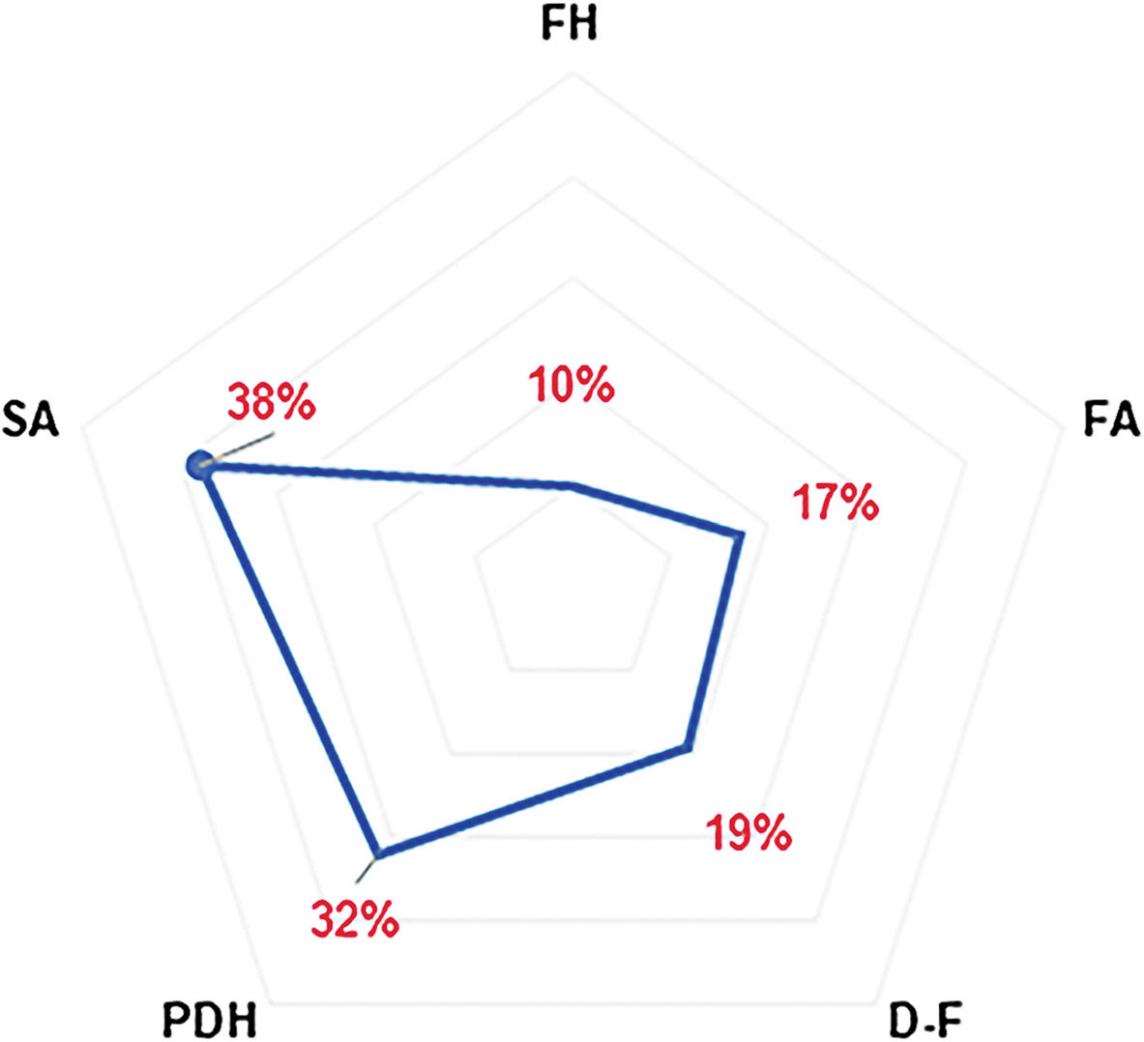
Rates of increase in different parameters after surgery. D-F, disc-to-facet distance; FH, foraminal height; PDH, posterior disc height; SA, segmental angle; FA, foraminal area

All the measured parameters increased significantly after the surgery (*p* < 0.01 for all) (Table 2). The change in the SA (average 37.58% ± 16.77%) was the most noticeable while the change in the FH (average 10.42% ± 6.34%) was the smallest (Fig. 3).

**Table 2:** Changes in contralateral intervertebral foramen parameters postoperatively

	Pre-operation	Post-operation	Average rate of increase	*p* value
FH (mm)	19.22 ± 2.53	21.20 ± 2.69	10.42% ± 6.34%	< 0.01
FA (mm^2^)	95.24 ± 15.32	111.37 ± 17.31	17.36% ± 9.48%	< 0.01
D-F (mm)	4.63 ± 1.47	5.46 ± 1.55	18.80% ± 8.52%	< 0.01
PDH (mm)	6.30 ± 1.23	8.24 ± 1.50	32.22% ± 18.92%	< 0.01
SA (degrees)	6.53 ± 2.09	8.87 ± 2.66	37.58% ± 16.77%	< 0.01

*D-F* disc-to-facet distance, *FH* foraminal height, *PDH* posterior disc height, *SA* segmental angle, *FA* foraminal area

### Correlations among the rates of change in various parameters of the contralateral IVF

Overall, the change in the PDH had the positive correlation with the change in the FH and FA (*p* < 0.01 for both), and no other significant correlation was confirmed (Table 3). Interestingly, however, the change in the SA had the negative correlation with the change in the FA in the o-group (*p* < 0.01) (Table 4) and had the positive correlation with the change in the PDH in the t-group (*p* < 0.05) (Table 5).

**Table 3:** Overall correlations among rates of change in contralateral intervertebral foramen parameters

	FH	FA	D-F	SA
PDH	0.516**	0.553**	0.131	0.228
SA	− 0.158	− 0.197	0.025	–

**p < 0.01

*D-F* disc-to-facet distance, *FH* foraminal height, *PDH* posterior disc height, *SA* segmental angle, *FA* foraminal area

**Table 4:** Correlations among rates of change in various parameters in the oblique group

	FH	FA	D-F	SA
PDH	0.660**	0.301	0.023	− 0.001
SA	− 0.297	− 0.596**	− 0.144	–

**p < 0.01

*D-F* disc-to-facet distance, *FH* foraminal height, *PDH* posterior disc height, *SA* segmental angle, *FA* foraminal area

**Table 5:** Correlations among rates of change in various parameters in the transverse group

	FH	FA	D-F	SA
PDH	0.276	0.793**	0.283	0.377*
SA	− 0.197	0.252	0.170	–

**p < 0.01; *p < 0.05

*D-F* disc-to-facet distance, *FH* foraminal height, *PDH* posterior disc height, *SA* segmental angle, *FA* foraminal area

### Comparisons between o-group and t-group

**Fig. 4:**
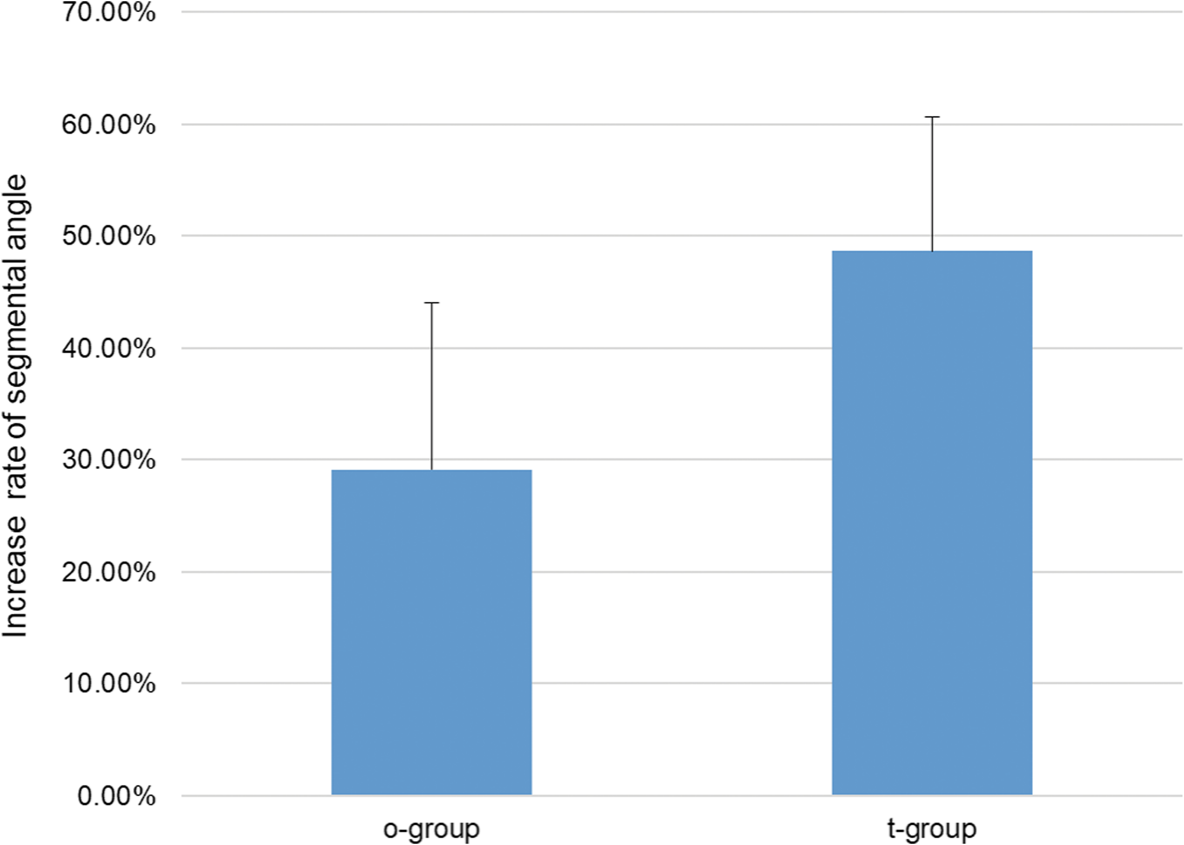
Comparison of the rate of increase in the segmental angle between the two groups

Between o-group and t-group, there was a significant difference in the change rate of SA (*p* < 0.01), while comparison of the other parameters showed no significant differences (Table 6). The mean SA degree increased by an average of 29.08% ± 14.93% in the o-group and 48.63% ± 12.01% in the t-group (Fig. 4). This difference remained significant after further correction for the age, gender, BMI, cage size, and fusion level (*p* < 0.01) (Table 7). The 6-month clinical outcomes of the two groups were also collected and analyzed. There was no significant difference in the improvement of the visual analog scale (VAS) and Oswestry disability index (ODI) scores between the two groups (Table 8).

**Table 6:** Comparison of rates of change in various parameters between transverse and oblique groups

	FH	FA	D-F	PDH	SA
*p* value	0.729	0.667	0.169	0.208	< 0.01

*D-F* disc-to-facet distance, *FH* foraminal height, *PDH* posterior disc height, *SA* segmental angle, *FA* foraminal area

**Table 7:** Multiple linear regression analysis with change in segmental angle as dependent variable

	β coefficient	*p* value
Age	0.040	0.675
Gender (male = 0, female = 1)	− 0.172	0.083
BMI	− 0.106	0.307
Segment (upper = 0, lower = 1)	0.269	0.006*
Cage size (small = 1, medium = 2, large = 3)	0.176	0.075
Cage orientation (oblique = 0, transverse = 1)	0.598	0.000*

*p < 0.01

*BMI* body mass index

**Table 8:** Comparison of clinical outcomes between transverse and oblique groups

	Pre-operation	3 months post-operation	6 months post-operation	*p* value*
VAS leg (o-group)	7.21 ± 1.03	2.38 ± 1.25	2.05 ± 0.97	0.186
VAS leg (t-group)	7.77 ± 1.04	2.13 ± 1.01	1.97 ± 0.72	–
VAS back (o-group)	5.85 ± 1.23	2.10 ± 0.94	1.85 ± 0.90	0.335
VAS back (t-group)	5.97 ± 1.52	2.53 ± 0.94	2.07 ± 0.74	–
ODI (o-group)	49.51 ± 9.65	23.41 ± 6.46	14.31 ± 4.32	0.966
ODI (t-group)	52.20 ± 10.09	21.20 ± 5.74	15.13 ± 4.84	–

*Comparison of the improvement rates of VAS and ODI between the two groups at 6 months postoperatively

*ODI* Oswestry disability index, *VAS* visual analog scale

## Discussion

Theoretically, IVS distraction via TLIF will produce two direct effects: an increase of DH and a change of SA. These effects were confirmed in our study. Overall, our outcomes demonstrated that an increase in the PDH resulted in a proportional increase in the FA and FH, while no significant correlations were found between the SA and other parameters. Therefore, it was reasonable to conclude that the increased PDH was a major factor responsible for the postoperative morphological changes of IVF. We also found a significant increase in the D-F, which could be approximately representative of the foraminal width. The increase in the D-F might result from either possible movement of the SAP or retraction of the annulus fibrosus. However, the SAP had a tendency to move towards the disc because of the increased lumbar lordosis; therefore, it was the retraction of annulus fibrosus that resulted in the D-F increase. This finding may help to explain a mechanism underlying the decompression of contralateral IVF, as the nerve root can be compressed by herniated discs in the IVF. Moreover, our results showed that neither the PDH nor SA had a significant correlation with D-F; in other words, it was not the more distraction of IVS, the more retraction of annulus fibrosus. This can be explained by the individual differences in the annulus fibrosus. Besides, the incomplete discectomy during TLIF procedure might also affect the retraction of the annulus fibrosus.

In our study, all cages were located at or close to the center of the IVS. By contrast, lateral cage placement in the IVS could cause the reduction of contralateral FH [14], and anterior cage placement could also decrease the contralateral foraminal size [7, 15]. It seems that middle cage placement would ensure satisfactory radiographic outcomes of the contralateral IVF. However, our results indicated that when the cage was placed obliquely in the IVS, the increase in the SA could decrease the contralateral FA. That is, the restoration of lumbar lordosis via TLIF has a negative impact on the enlargement of contralateral intervertebral foramen. This may be due to the unsymmetrical cage location in the IVS (anterior on the contralateral side and posterior on the ipsilateral side), which cannot maintain the stability between the bilateral posterior elements. In addition, when comparing these parameters, we should also consider the effects of different cage shapes. The widely used wedge-shaped or lordotic cages are designed to fit the vertebral endplate more tightly. However, a potential disadvantage of these curved cages might be that they can reduce the foraminal size because of the “additional” increase in segmental lumbar lordosis [7, 16, 17]. By comparison, the cage used in our study had a straight body shape, and no decreasing parameters of the contralateral IVF were found.

It should be noted that the extent of the increase in or restoration of the SA that might lead to adverse results has not been established. Jang et al. found that the rate of increase in the SA in patients with contralateral radiculopathy was obviously higher than that in asymptomatic patients [17]. However, another recent study reported that the change in the SA was not a risk factor for postoperative contralateral radiculopathy [18]. According to our radiographic outcomes, whether the increase in the SA was beneficial or harmful to the contralateral foraminal size seemed to depend on the cage insertion orientation and the final cage location within the IVS.

The major limitation of this study is the lack of long-term follow-up data. Cage subsidence or loss of lordosis was not taken into account. However, our research is still valuable as it is the first study to investigate retraction of the annulus fibrosus and morphological changes of the contralateral IVF regarding cage insertion orientation. Our findings provide insights into the possible mechanisms underlying indirect decompression of the contralateral IVF via unilateral TLIF. Moreover, despite the lack of a long-term clinical data, our study provided two valuable findings: First, the transverse cage insertion can achieve more restoration of segmental lordosis, which is very important in biomechanical and clinical aspects [19]. Second, the oblique cage insertion method has a negative impact on the enlargement of contralateral intervertebral foramen due to the unsymmetrical cage location within the IVS.

## Conclusions

Quantitative parameters of the contralateral IVF increased significantly after unilateral TLIF, and among these changes, the retraction of the annulus fibrosus was relatively prominent. Compared to the oblique cage insertion method, transverse cage insertion in the center of the IVS could achieve more restoration of segmental lumbar lordosis without decreasing the contralateral foraminal dimensions. Notably, oblique cage insertion method could impair the effect of indirect decompression.

## Abbreviations

BMI: Body mass index
D-F: The disc-to-facet distance
FA: Foraminal area
FH: Foraminal height
IVF: Intervertebral foramen
IVS: Intervertebral disc space
ODI: Oswestry disability index
PDH: Posterior disc height
SA: Segmental angle
SAP: Superior articular process
TLIF: Transforaminal lumbar interbody fusion
VAS: Visual analog scale
